# World-record envelopes: The speed-distance trade-off in racing events

**DOI:** 10.1371/journal.pone.0359859

**Published:** 2026-10-07

**Authors:** John Yinger

**Affiliations:** Professor Emeritus of Economics and Public Administration, Syracuse University, Syracuse, New York, United States of America; Universidade do Porto, PORTUGAL

## Abstract

The concept of a mathematical envelope, which is widely used in economics, can be applied to the relationship between world-record racing speeds and event distances. This paper derives and estimates world-record envelopes for running, freestyle swimming, and speed skating and shows how these envelopes are related to the underlying family of individual performance curves. It also compares world-record envelopes for men and women and shows that the world-record envelope in men’s running has shifted upward over time but has not changed shape in 120 years. Finally, this paper shows that the speed-distance envelope reflects not only the trade-off between speed and distance for individual athletes but also the change in this trade-off as one moves from one athlete to another. The failure of existing studies to recognize the second effect leads to a misinterpretation of the observed link between world-record performances and human physiology. The paper also shows how this problem can be avoided by estimating individual performance curves using the personal best times of elite athletes.

## 1. Introduction

In his classic book on the American astronauts, Wolfe reports that Navy pilots often talk about their flying experiences.

One of the phrases that kept running through the conversation was “pushing the outside of the envelope.” The “envelope” was a flight-test term referring to the limits of a particular aircraft’s performance, how tight a turn it could make at such-and-such a speed, and so on. “Pushing the outside,” probing the outer limits, of the envelope seemed to be the great challenge and satisfaction of flight test (Wolfe [1], p. 12).

This paper explores the efforts of elite athletes to push out their own (less dangerous) envelopes. This approach in this paper sheds light on the trade-off between speed and distance facing elite athletes in running, swimming, and skating and sets the stage for an analysis of the factors that influence world-record performances.

The focus in this paper is on the speed-distance tradeoff in racing world records. Building on the pioneering work of A.V. Hill [2] and Monod and Scherrer [3], many recent studies address this topic, although their functional forms and interpretations vary widely. One group of studies, which includes Grubb [4], Katz & Katz [5], Kuper & Sterken [6], Riegel [7], and Roy et al. [8], regresses racing speed, *S*, or racing time, *T*, on a function of event distance, *D*, without applying their model to individual racers. A so-called power function regression of the log of *S* on the log of *D* appears in Grubb [4] and Riegel [7]. Except for Riegel [7], these studies all give their models a physiological interpretation. As Riegel [7, p. 290] puts it: “I have tried to avoid speculation regarding physiological reasons for the effects noted in this article, preferring only to describe the reality as I have seen it.”

Another group of studies, including Péronnet & Thibault [9], Emig & Adams [10], Mulligan, Adam, & Emig [11], each build an elaborate model of individual racing performance based on physiological principles and then apply that model to the average performance of world record holders. This strategy is inconsistent. Mulligan, Adam, & Emig [11] recognize, for example, that “many parameters can be different among individuals,” but they then explain that their “model depends on the four independent parameters … that characterize a group of runners (for example world record holders) or individual runners.” A similar statement appears in Emig & Adam [10]. Substituting average values into an individual’s performance curve does not provide a compelling picture of the time-distance trade-off.

Finally, a few studies, including Blythe & Király [12], Drake et al. [13] and Vandewalle [14], estimate speed-distance tradeoffs based on the best times of individual runners in a set of events. This approach is equivalent to estimating individual performance curves, which are part of an envelope specification. See Section 3.3. Moreover, this approach can illuminate the impacts on racing performance of individual runners’ physiological traits and of racing conditions. See Section 4.

This paper uses a different approach, namely, to specify the time-distance trade off using the mathematical concept of an envelope. A mathematical envelope for a family of curves is another curve that is tangent to every member of the underlying family. Moreover, an “upper” envelope is the set of highest points for the “y” variable at each value for the “x” variable. The highest point at a given “x” is associated with a single member of the family of curves. A lower envelope is the set of lowest points for each “x.” For the application of these concepts to the athletics races considered in this paper, think of each member of a family of curves as the set of best performances for a given athlete in a series of related events of different lengths. The associated lower envelope is the set of fastest times (= lowest values with time on the vertical axis), and the upper envelope is the set of highest speeds.

This paper focuses exclusively on upper envelopes. Expressing world records in terms of speed, not time, makes the world-record envelopes more intuitive and easier to understand. As discussed in Section 2, this approach has some methodological advantages over a time-focused alternative.

This paper estimates world-record envelopes—the relationship between world record speeds and distance—for running, freestyle swimming, and ice skating. The results are used to shed light on the trade-off between speed and distance that faces elite athletes. These envelopes are compared across sports, years, and genders. Although estimates of this relationship have appeared in the literature, no previous study of the topic recognizes that it is estimating an envelope, which is mathematically connected to the underlying family of individual performance curves. This limitation leads to misconceptions, discussed below, in many studies.

The envelopes estimated in this paper are continuous functions that estimate the highest observed speeds of record-setting athletes in races of different lengths. They are not simply plots of world records at a point in time, although such plots are referred to as envelopes in the title of Masood [15] and are examined by Berthelot et al. [16] and Kruse et al. [5]. Instead, the envelopes in this paper reflect not only differences in the physiological demands of different events but also differences in the abilities of different athletes.

The concept of an envelope appears in economics. Every economist learns that the long-run average cost curve is the envelope of a family of short-run cost curves with different plant sizes. A large literature examines housing hedonic functions, which indicate the impact of neighborhood amenities on housing prices, and which are the envelope of underlying household bid functions for those amenities. See Nguyen-Hoang & Yinger [17] and Yinger [18].

These studies are all constrained by the fact that a single point is observed for each individual or firm, namely, the point at which that individual’s bid function (or that firm’s short-run cost function) is tangent to the envelope. Application of these concepts to world records in running has the advantage that multiple points, defined by personal best times in various events, can be observed for each elite athlete. This feature makes it possible to estimate the individual performance curves that combine to create the world-record envelope. The analysis in this paper is limited to racing world records, but it might also shed light on other human endeavors in which the analog to individual performance curves can be observed.

## 2. Materials and methods

### 2.1. Individual performance curves and world-record envelopes

A hypothetical example of the relationship between a world-record envelope and the family of individual performance curves on which it is based is illustrated in Fig 1. Speed in miles per hour (mph) or kilometers per hour (km/h) is on the vertical axis and distance (in miles or kilometers) is on the horizontal axis. A world record corresponds to a point of tangency between an individual performance curve and the envelope. Overall, the shape of the envelope reflects two factors: the speed-distance tradeoffs for individual record holders and the change in the speed-distance tradeoff as one moves from one athlete to another.

**Fig 1:**
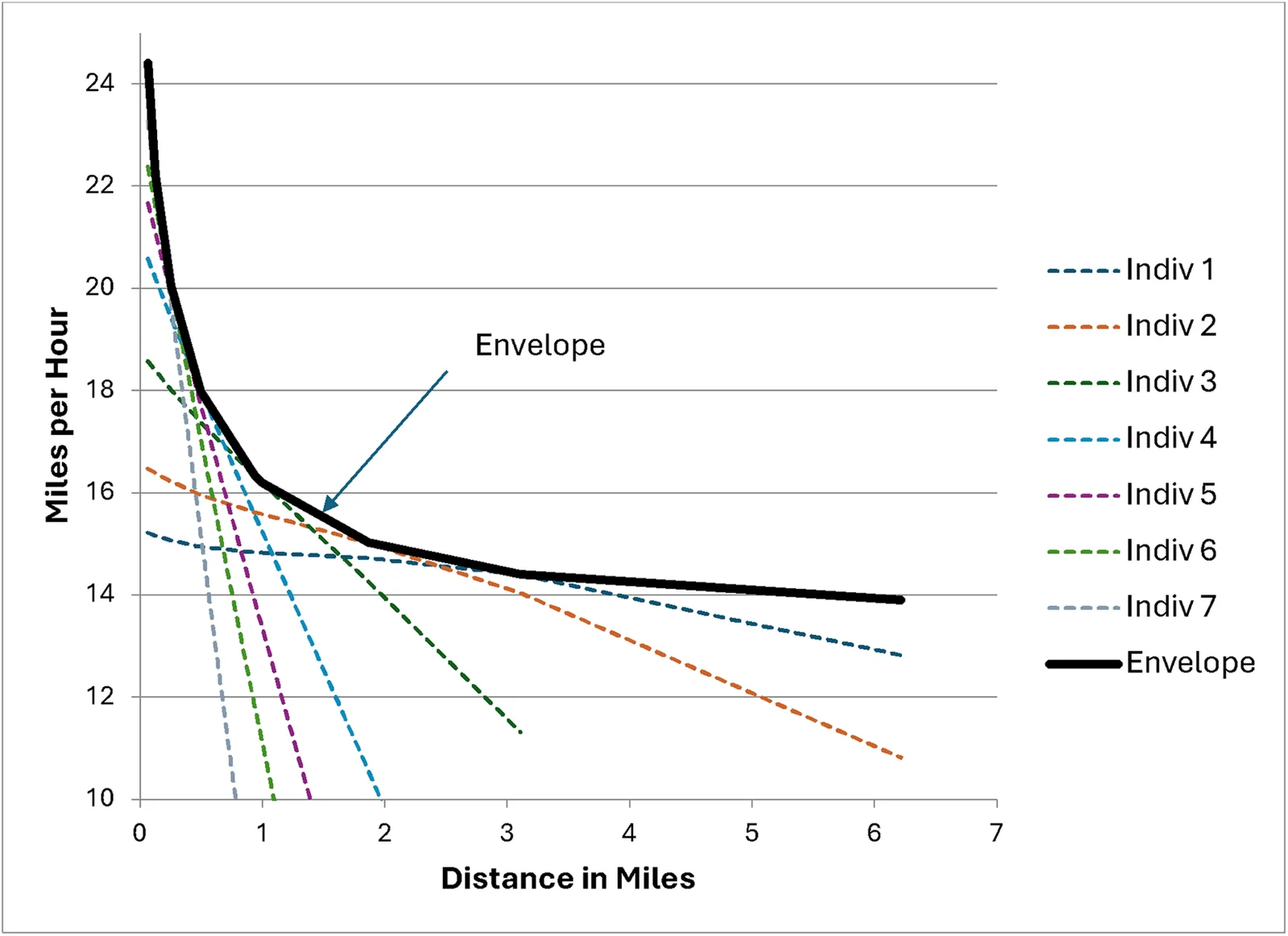
Individual performance curves and the world-record envelope.

The analysis in this paper requires a form for the envelope that applies to racing world records, which I call the world-record envelope. A form of this type provides insight into the speed-distance tradeoff facing elite athletes and shows how that tradeoff varies across sports, over time, and between men and women.

Several possible envelope forms are described in Table 1. If speed, *S*, is the dependent variable for the individual performance curve in the first row, for example, the associated envelope is a quadratic function of distance, *D*. Fig 1 is based on the case in which *S* is set equal to the forms in the last row of this table. Other cases in this table are considered below.

**Table 1:** Individual performance curves and the world-record envelope.

Form	Individual Performance Curve	World-Record Envelope
Quadratic	$\alpha +\delta \beta +\delta^{\text{2}}\gamma +\delta \theta D$	$\alpha -\left(\frac{\beta^{\text{2}}}{4\gamma }\right)+\left(\beta \theta \right)D-\left(\frac{\theta^{\text{2}}}{4\gamma }\right)D^{\text{2}}$
Log	α+δβD+γlog{δ}	$\left(\alpha -\gamma +\gamma log\left\{\frac{-\gamma }{\beta }\right\}\right)-\gamma log\left\{D\right\}$
Log^2^	$\alpha +\delta^{\text{2}}\beta +\delta \gamma log\left\{D\right\}$	$\alpha -\left(\frac{\gamma^{\text{2}}}{4\beta }\right){\left(log\left\{D\right\}\right)}^{\text{2}}$
IHC	$\alpha +\beta -\delta \beta D+\beta log\left\{\delta \left(1+\sqrt{1+D^{\text{2}}}\right)\right\}$	$\alpha +\beta log\left\{\frac{1+\sqrt{1+D^{\text{2}}}}{D}\right\}$

Notes: An envelope for a family of curves *F*{*y*, *x, δ*} is an equation that satisfies *F*{*y, x, δ*} = 0 and d*F*/d*δ* = 0, where d denotes a derivative. The envelope forms in this paper can all be derived from a family of curves expressed as *S* + IPC = 0 [or log{*S*} + IPC = 0], where *S* is speed (in miles per hour, mph, or kilometers per hour, km/h) and IPC is an “individual performance curve.” The IPCs in column one are functions of *D,* which is distance (in miles or kilometers), and *δ*, which is a parameter that varies across athletes. The other Greek letters are constants. The row labels refer to the form of the envelope in the last column. Row 2 with log{*S*} in the equation family formula is called a power function, which is widely used in the literature. This paper focuses on the last row with *S* in the equation family formula. IHC stands for “inverse hyperbolic cosecant.” All logs in this table are natural logs.

The approach in this paper differs from the approach in previous articles in four main ways. First, many previous articles use the same specification to estimate the relationship between *S* and *D* for individuals (which I call an individual performance curve) and the relationship between *S* and *D* across world records (which I call the world-record envelope). Although these two equations are obviously related, the examples in Table 1 make it clear that they have different specifications. It is not appropriate, for example, to estimate a power function for both the individual performance curves and the envelope. Instead, one must either assume the envelope is a power function with log{*S*} as the dependent variable and use the formulas in row two of Table 1 or assume that the individual performance curve is a power function and use the formulas in the third row. Estimates using Table 1 as a guide are provided in Section 3.3. A failure to recognize the distinction between individual curves and their envelope is not unique to world-record envelopes. As shown by Yinger and Nguyen-Hoang [18], this misconception also arises in the case of housing hedonics.

Second, this paper uses hypothesis tests to determine the best form for the world-record envelope. Some previous studies are clever, but they do not determine whether their specification has more explanatory power than any other.

Third, unlike most of the literature, this study explains that individual performance curves—but not the world-record envelope—are appropriate for testing hypotheses about human physiology. The world-record envelope reflects both the speed-distance tradeoff for individual athletes, which depends on human physiology, and the shift from one athlete to another, which does not reflect the physiology of any athlete. See Section 3.4.

Finally, this study recognizes a “start-up” effect in most racing events and examines several methods to account for its impact on world records. One such method is to limit the sample to longer races, which do not have a large start-up effect. Some previous studies of running discard observations below 1,000 meters or even 1,500 meters based on the argument that the demands on a human body are different in sprints and longer races. As explained above, however, an envelope alone cannot measure different demands on a given human’s body at different race lengths. Moreover, as shown in Section 3.3, dropping only the 100 meter dash leads to an envelope that closely tracks world record speeds at all other distances.

### 2.2. Estimating world-record envelopes

To find a form for the world-record envelope, I begin with the self-evident assumption that world-record average racing speed is a function of an event’s distance. Building on this assumption, I then find the algebraic form for the world-record envelope that has the highest explanatory power. To carry out this step, I regress speed on distance using a variety of functional forms and select the form with highest R-squared.

The data for this step, namely, times and speeds for 13 running events, are presented in Table 2. Except for the last two, which are road races, these events are run on an outdoor track. Indoor events and events that are rarely staged are not included in this table. Rarely staged events are omitted because they have lower prestige and, in most cases, lower prize money so they may not attract the best runners. For evidence on this point, see Lynch and Zax [20] and Kruse et al. [21]. Women’s records are based on women-only events.

**Table 2:** World records in running, November 2024.

Race	Distance (Miles)	Men		Women	
		WR Time	WR Speed (mph)	WR Time	WR Speed (mph)
100 m	0.06214	9.58s	23.3501	10.49s	21.3245
200 m	0.12427	19.19s	23.3136	21.34s	20.9648
400 m	0.24855	43.03s	20.7943	47.6s	18.7978
800 m	0.49710	1mi 40.9s	17.7359	1mi 53.3s	16.6315
1,000 m	0.62137	2mi 12.0s	16.9465	2mi 29.0s	15.0130
1,500 m	0.93206	3mi 26.0s	16.2884	3mi 49.0s	14.6525
1 mile	1.00000	3mi 43.1s	16.1363	4mi 07.6s	14.5396
2000 m	1.24275	4mi 43.1s	15.8032	5mi 19.7s	13.9940
3000 m	1.86412	7mi 16.6s	15.3355	8mi 06.1s	13.3118
5,000 m	3.10686	12mi 35 4s	14.8260	14mi 21.0s	12.8994
10,000 m	6.21373	26mi 11.0s	14.2390	18mi 51.7s	12.5459
½ marathon	13.10940	57mi 03.0s	13.2539	1h 5mi 16.0s	12.0516
marathon	26.21880	2h 35.0s	13.0460	2h 16mi 16s	11.5445

Source: International Olympic Committee [19]. Notes: “m” stands for meter; “WR” stands for world record; “h” stands for hours; “mi” stands for minutes; “s” stands for seconds; “mph” stands for miles per hour. Speed in kilometers per hour (km/h) is speed in mph multiplied by 1.60934. Records as of November 15, 2024.

Speed-distance regressions based on these data with several different specifications are presented in Table 3. These specifications include the power function used in several previous studies (and included in the second row of Table 1), and the inverse hyperbolic cosecant (included in the last row of Table 1). Several specifications in Table 3 have high explanatory power, but the clear winner, with an R-squared of 0.974, is a regression with the explanatory variable specified as an inverse hyperbolic cosecant of distance. Moreover, a Davidson-MacKinnon test, which compares two non-nested specifications, rejects the second-ranked TA specification in favor of the IHC specification. (See the supplemental data file.) As shown below, the IHC specification also works well with events other than men’s running. Moreover, this form makes it possible to identify the underlying family of individual performance curves for which it is the envelope, namely, the curves in the last row of Table 1. We return to this topic in Section 3.3.

**Table 3:** Alternative forms for the world-record envelope.

	IHC	TA	Power	Log	CS	SE	Quadratic
Var1	3.1377	-9.6123	-0.116	-1.847	0.6498	7.3487	-0.969
t-stat	20.13**	-14.14**	-11.96**	-8.86**	5.87**	5.66**	-2.54**
Var2							0.0287
t-stat							1.92**
Constant	13.4856	23.4476	16.986	17.2967	15.2756	13.0693	19.0867
t-stat	56.52**	45.95**	62.1**	49.16**	26.71**	15.24**	19.34**
R-sq	0.9736	0.9479	0.9218	0.8772	0.758	0.7445	0.5134
F-stat	205.5465**	102.7427**	67.4706**	41.8584**	19.5987**	18.3737**	5.2754**

Notes: Regressions for 13 events in men’s running in 2020. The dependent variable is world-record speed in mph. Explanatory variables are functions of distance in miles using the indicated forms. “WR” stands for world record; “IHC” stands for inverse hyperbolic cosecant; “TA” stands for hyperbolic tangent; “CS” stands for hyperbolic cosecant; “SE” stands for hyperbolic secant. The power function is linear in logs but is estimated without logs using nonlinear least squares to make the R-squared comparable to the ones for the other equations. The data come from Wikipedia [22]. The symbol ** (*) stands for significance at the 5% (10%) level.

## 3. Results: World-record envelopes for running, swimming, and speed skating

World-record envelopes for running, freestyle swimming, and speed skating are presented in Table 4. These envelopes are estimated using the form in row 4, column 2 of Table 1. The coefficient of the distance-based term is highly significant in every case. Moreover, all the regressions have high explanatory power, although the R-squared is lower for speed skating than for the other sports. In addition, this table reveals that distance has a much smaller impact on swimming speed, and a slightly smaller impact on skating speed, than it does on running speed.

**Table 4:** Estimated world-record envelopes.

	Running		Swimming		Skating	
	Men	Women	Men	Women	Men	Women
Constant	13.489	11.96	3.276	3.202	30.115	27.511
t-stat	56.625**	58.554**	19.42**	22.854**	38.678**	41.064**
IHC	3.136	2.964	0.452	0.330	2.288	2.264
t-stat	20.155**	21.406**	7.293	6.420**	3.032**	3.487**
R-sq	0.987	0.974	0.930	0.912	0.697	0.752
F-stat	420.309**	208.752**	27.5352**	21.6812**	5.42211**	6.92296**
Observ	13	13	6	6	6	6

Notes: Regressions with speed (mph) as the dependent variable and the inverse hyperbolic cosecant (“IHC”) of distance (in miles) as the explanatory variable. The running data are for 2024. See Table 2. The swimming and skating data are for 2020 and come from Wikipedia [23]. Results for distance in kilometers are similar. See the supplemental data file. The symbol ** (*) stands for significance at the 5% (10%) level.

Fig 2 presents world records and world-record envelopes for men’s and women’s running. This figure is based on all 13 running events but is truncated for readability.

**Fig 2:**
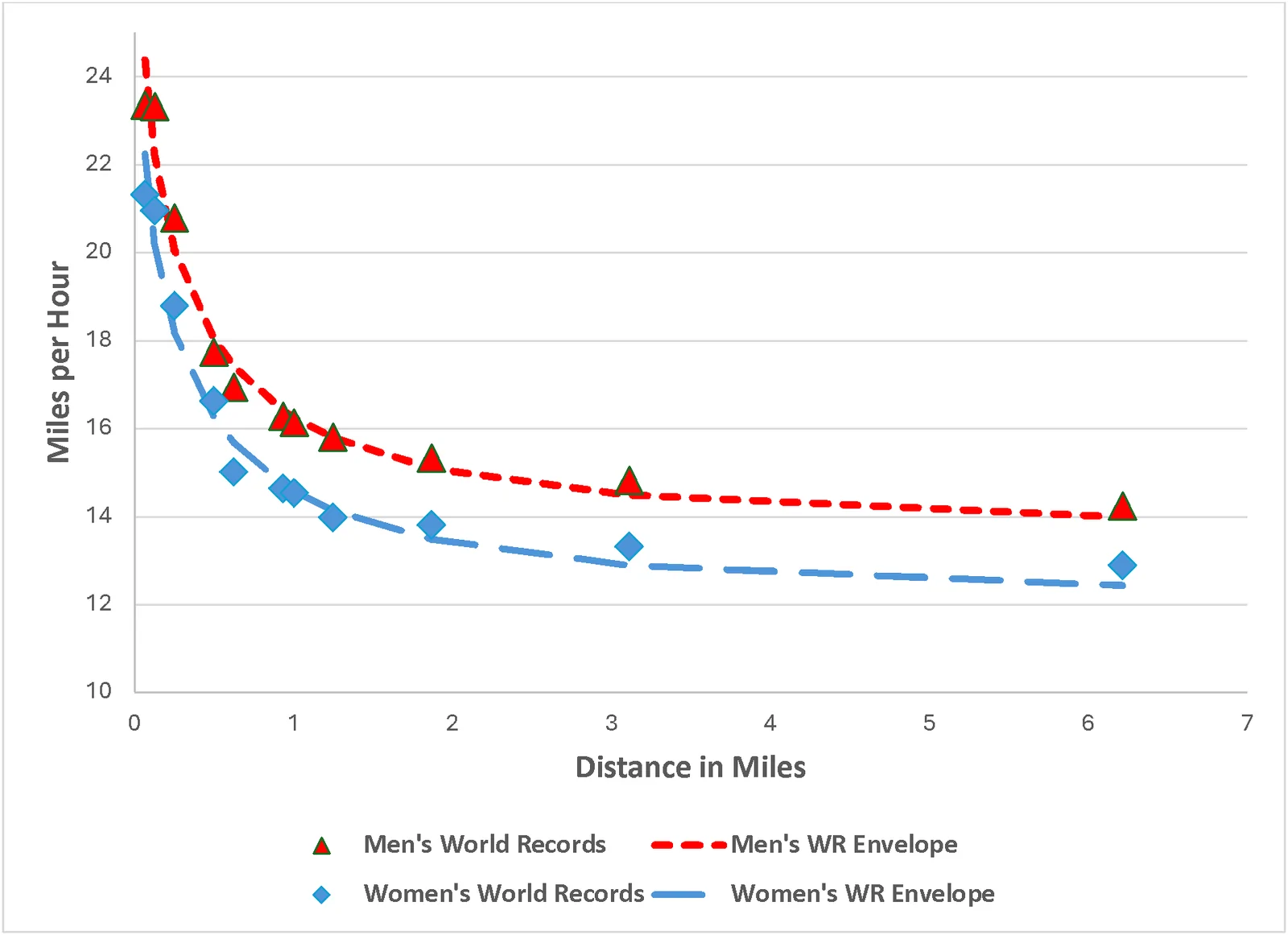
World-record envelopes, men’s and women’s running, 2024.

This figure confirms that the world-record envelope provides a close approximation to world records. Moreover, the men’s and women’s world-record envelopes have similar shapes. The intercepts are smaller for women than for men, but the differences are not statistically significant. The slopes are also smaller for women than for men and two of the differences (for running and skating) are significant. See Table 4.

### 3.1. The speed-distance tradeoff

Further information on the speed-distance tradeoff appears in Table 5. This table lists running events by distance and shows the increase in speed required to maintain a world record performance when moving up one row in the table, that is, when moving to a shorter event. A male runner who matches the world record speed for 800 meters, for example, would have to increase his speed by about 3.0 miles per hour to match the world record speed for 400 meters.

**Table 5:** The speed-distance trade-off in world records for running.

Comparison Event	Event	Cut in Distance (in Miles)	Required Increase in Speed (mph)	
			Men	Women
200 m	100 m	0.06214	0.0365	0.3597
400 m	200 m	0.12427	2.5194	2.1669
800 m	400 m	0.24855	3.0583	2.1663
1,000 m	800 m	0.12427	0.7894	1.6185
1,500 m	1,000 m	0.31069	0.6581	0.3606
1 mile	1,500 m	0.06794	0.1521	0.1129
2000 m	1 mile	0.24275	0.3331	0.5456
3000 m	2000 m	0.62137	0.4677	0.1886
5,000 m	3000 m	1.24275	0.5096	0.4936
10,000 m	5,000 m	3.10686	0.5870	0.4124
1/2 marathon	10,000 m	6.89567	0.5596	0.8479
marathon	1/2 marathon	13.10940	0.6334	0.5071

Notes: This table shows the increase in speed needed to stay on the world-record envelope when moving from the comparison event to the indicated event. It is based on Table 4.

For all the races longer than 800 meters, the required increase in mph to maintain a world-record pace for a shorter event is less than 0.8. This is a surprising regularity, because the change in distance ranges from 13.1 miles for a shift from the marathon to the half marathon to 0.124 miles for a shift from 1,000 meters to 800 meters.

The first row of Table 5 indicates that the world record speed for 100 meters is not much faster than the record speed for 200 meters. This start-up effect also appears in Fig 2, although it is difficult to detect there because the triangles indicating the world record speeds for the 100 and 200 meter dashes are overlapping. This effect reflects the reality of starting, that is, of getting out of the blocks. The record speeds for a 100 meter dash, 23.35 mph (37.58 km/h) for men and 21.32 mph (34.31 km/h) for women, are only slightly faster than the record speeds for a 200 meter dash, 23.31 mph (37.51 km/h) for men and 20.96 mph (33.72 km/h) for women. Moreover, the record speed for the 100 meter dash is greater than the record speeds for the 50 meter dash, 20.12 mph (32.38 km/h) for men and 18.77 mph (30.21 km/h) for women, which is an indoor event not included in Fig 2. In 2009, Usain Bolt’s speed was clocked at 27.78 mph (44.71 km/h) between meters 60 and 80 of a 100 meter race (Wikipedia [23].)

The start-up effect does not appear in the forms in Table 1, so the world-record envelopes in Table 4 are less accurate for the shortest races than for the longer ones. Several ways to address this issue are available, depending on a study’s objective. A study providing a broad look at world records, such as this one, can proceed with the forms in Table 1 recognizing that they may overstate speeds to some degree for the shortest races. Studies that focus on the envelope itself, without estimating the underlying individual performance curves, can introduce a start-up adjustment to the envelope regression. It takes a sprinter about 30 meters to get up to speed (Healy, Kenny, and Harrison [24]). Thus, a second approach is to define a variable equal to the ratio of 30 meters to the race distance and to include this variable in the envelope regression. A third approach is to drop the observations for the shortest races.

Fig 3 compares these approaches for the case of men’s running. The broad-brush approach, which is also presented in Fig 2, overstates speeds for the 100 meters and understates speeds for the 200 and 400 meters races, although the degree of misstatement is not large in any case. Adding the start-up variable leads to more accurate predictions, but it breaks the mathematical link between the envelope and the underlying individual performance curves described in Table 1. Finally, dropping the 100 meter dash from the regression also leads to more accurate predictions at the expense of some loss of information.

**Fig 3:**
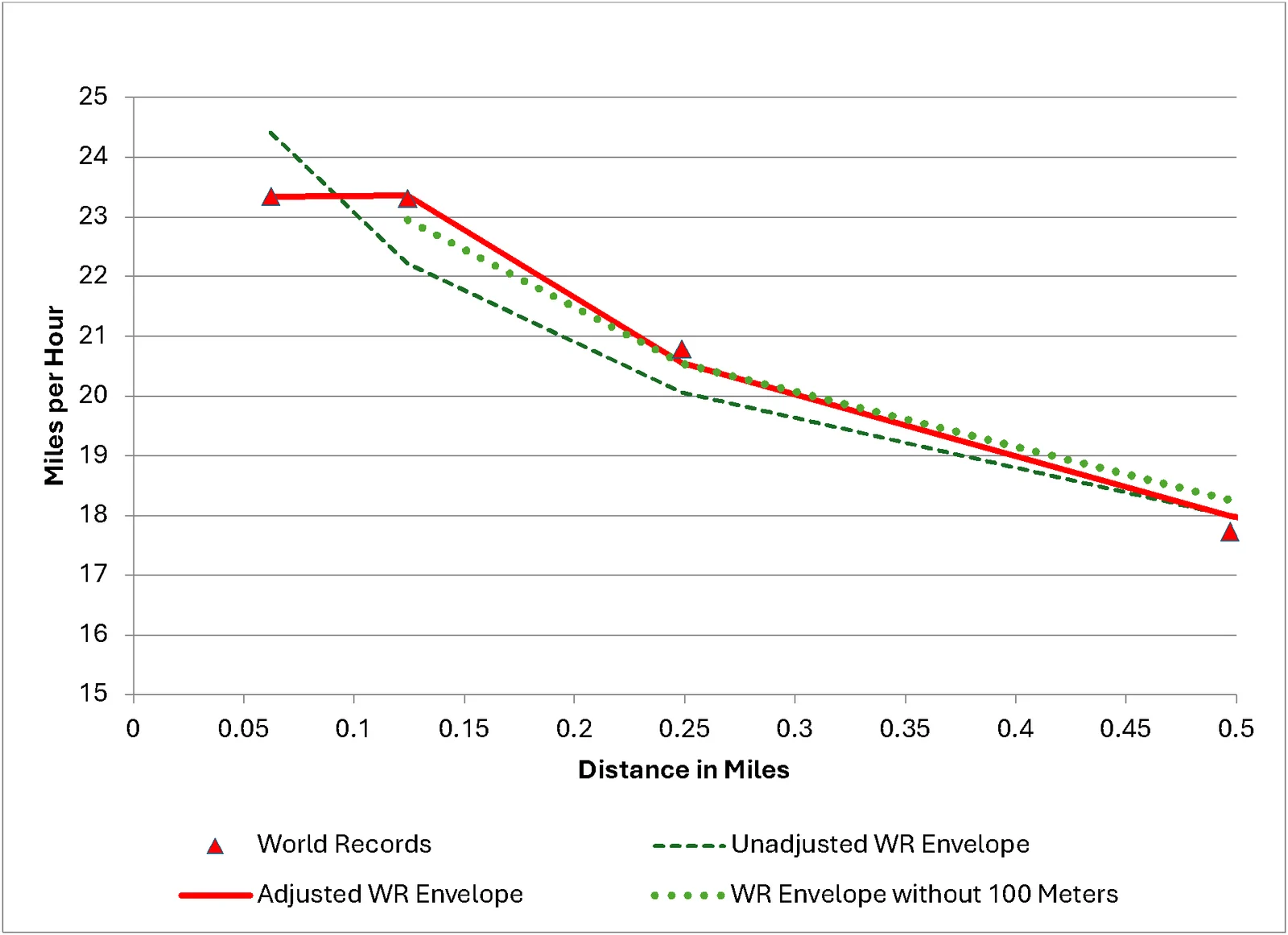
Accounting for the start-up effect in men’s running.

Fig 4 and Fig 5 show estimated world-record envelopes for freestyle swimming and speed skating, respectively. In both figures, the shapes of the world-record envelopes are virtually the same for men and women, with somewhat slower speeds for women than for men. The results in these two figures are reinforced by the regression results in Table 4.

**Fig 4:**
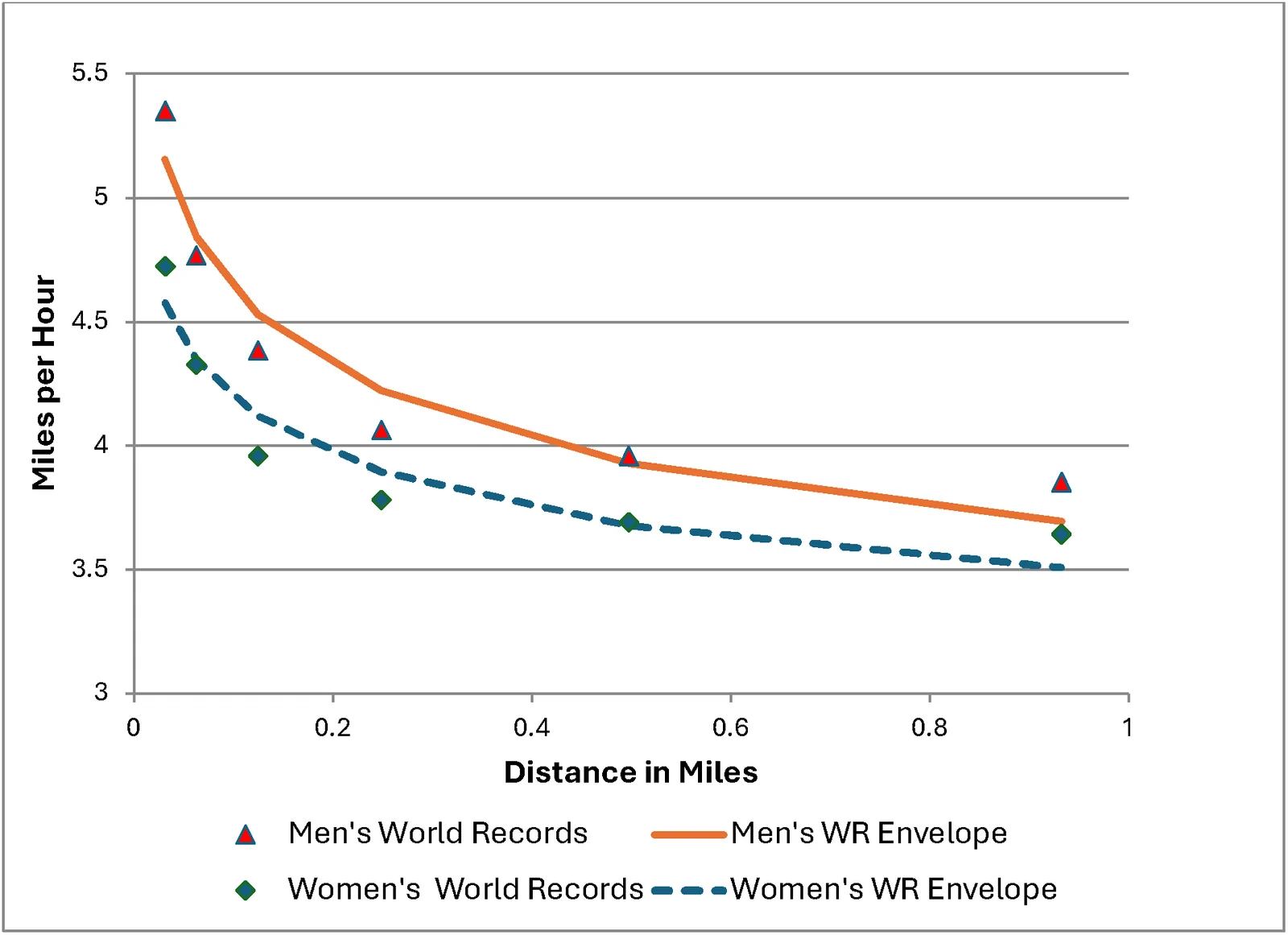
World-record envelopes, men’s and women’s freestyle swimming 2020.

**Fig 5:**
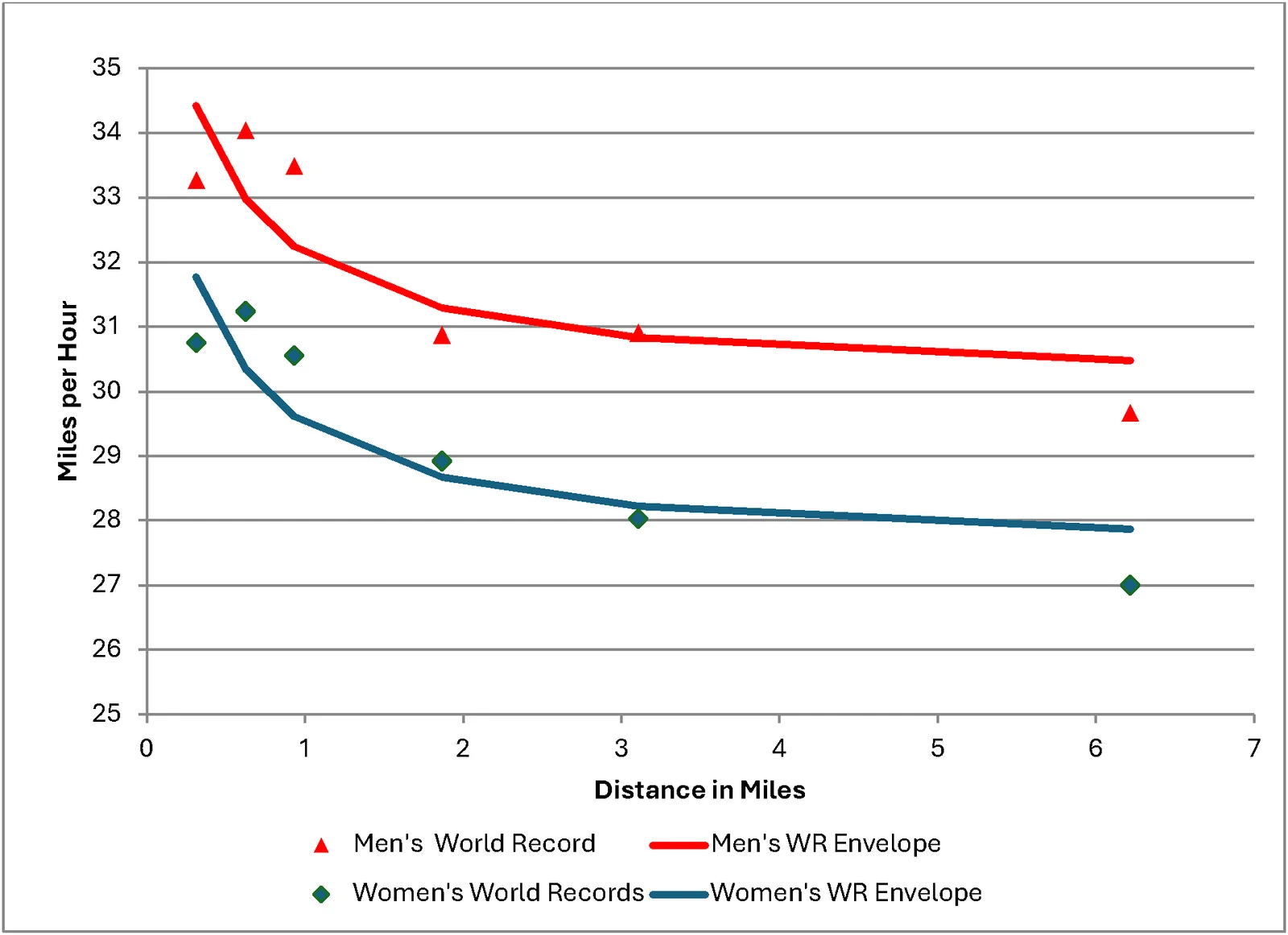
World-record envelopes, men’s and women’s speed skating, 2020.

Fig 4 also shows that the start-up effect is even greater in speed skating than in running. In fact, the men’s and women’s record speeds are actually higher over 1000 meters (34.05 mph; 54.80 km/h) than over 500 meters (33.28 mph; 53.56 km/h), and the record speed for men at 1500 meters (33.49 mph; 53.90 km/h) also exceeds their record 500 meter speed. Apparently, it takes skaters a while to get up to cruising speed. In contrast, no start-up effect is apparent for freestyle swimming; the diving start appears to bring. swimmers up to speed right away.

### 3.2. Envelope progressions over time

The envelope approach can be applied to world records in different years. This step makes it possible to determine not only whether and by how much record speeds have increased, but also whether the speed-distance trade-off has changed. Do modern training methods or running shoes boost speed at a given distance or distance at a given speed? Previous analysis of this topic using different forms appears in Katz and Katz [5] and Kruse et al. [21]. Some previous studies (Berthelot et al. [16], Kuper & Sterken [6], Nevill & Whyte [9], Nevill et al. [25]) use the time progression to project the likely future path of these records. No such forecast is presented here.

Fig 6 and Table 6 provide answers to these questions. The world-record envelope has shifted upward over the years. This point is clear in Fig 6 and is documented by the significant, growing year effects across the first row in Table 6. These results give no indication, however, that the trade-off between speed and distance has changed significantly over the last 120 years—at least in the case of men’s running. Indeed, the visual evidence in Fig 5 and the insignificance of the interaction terms between year and the distance variable in Table 6 indicate that the shape of the envelope has not changed since 1900. Table 4 and Fig 2 show that the shape of the world-record envelope for women’s running in 2020 is similar to the men’s counterpart. However, Table 6 and Fig 6 cannot be replicated for women, because long-distance races for women—and associated world records—were not all sanctioned until the 1970s. See Lovett [26].

**Table 6:** Time progression in world-record envelope, men’s running.

		Interactions with Year		
		1940	1980	2020
Intercept	10.6261	1.1494	2.3412	2.8415
t-stat	23.6862**	1.8117*	3.6902**	4.4788**
IHC	3.2716	-0.019	-0.1439	-0.1105
t-stat	12.882**	-0.053	-0.4007	-0.3077
R-sq	0.9608			
F-stat	98.0408**			
Observ	36			

Notes: This table regresses men’s world-record speed in 13 events on the IHC (inverse hyperbolic cotangent) term defined by Table 1 with year fixed effects and interactions between those effects and IHC. The baseline is 1900. The symbol ** (*) stands for significance at the 5% (10%) level.

**Fig 6:**
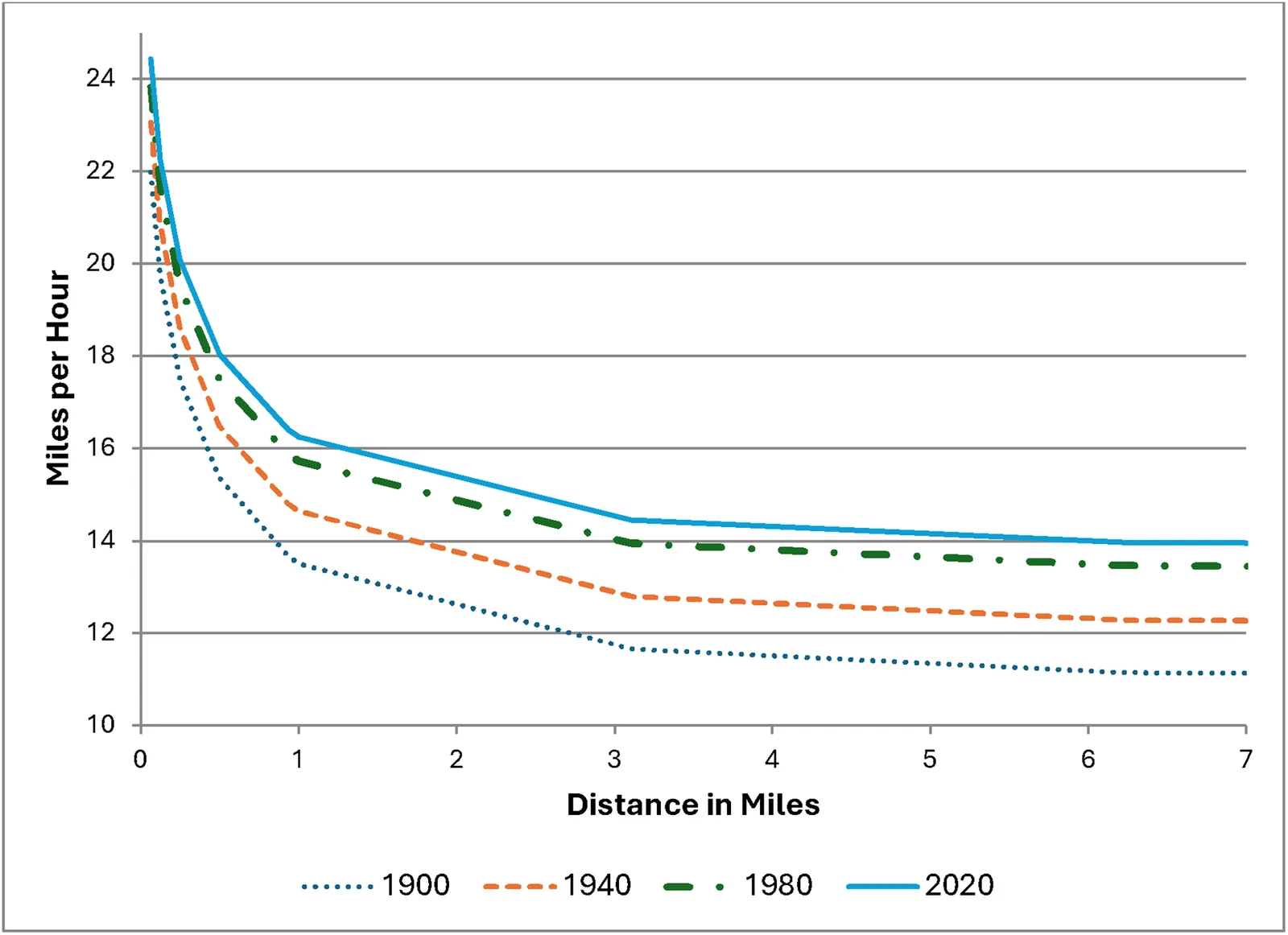
World record progression, men’s running.

### 3.3. Individual performance curves and world-record envelopes

Fig 2, Fig 4, and Fig 5 show the envelopes of underlying individual performance curves. In theory, an envelope should be tangent with a single performance curve at any given distance. In practice, however, some athletes are so gifted that they set the world record in more than one event. Moreover, the linkages between performance curves and envelopes may be affected by random events. Nevertheless, the performance curve-envelope logic is still approximately right and provides some valuable formal structure for an analysis of athletic contests.

Table 7 shows the estimated individual performance curves of male runners who each hold two world records: Usain Bolt (100 and 200 meters), Hicham El Guerrouj (1500 meters and the mile), Jakob Ingebrigtsen (2000 and 300 meters), and Joshua Cheptegei (5,000 and 10,000 meters). David Rudisha, the 800-meter world-record holder, is also included. These individual performance curves are based on the personal best times of each runner as of 2024 and the formula in row 4, column 1 of Table 1.

**Table 7:** Individual performance curves, men’s running, 2024.

	Bolt	Rudisha	El Guerrouj	Ingebrigtsen	Cheptegei
Constant	379.2610	48.0793	26.1989	19.0003	16.3813
t-stat	4.8965	1.1830	5.7139	47.6241	42.3840
Distance	19.0598	-2.1428	3.2878	0.2032	0.0338
t-stat	2.1892	-0.1833	1.6176	2.7328	1.0060
Second Term	-514.3750	-39.3629	-15.1248	-3.4670	-1.2807
t-stat	-4.5714	-0.6378	-2.0236	-7.2151	-3.7306
R-sq	0.9916	0.9758	0.9751	0.9869	0.9593
Observ	5	5	7	9	6
Implied parameter values					
α	-801.4205	##	18.2415	12.6319	13.0070
β	-514.3750	-39.3629	-15.1248	-3.4670	-1.2807
δ	-0.0371	0.0544	-0.2174	-0.0586	-0.0264

Note: This table presents individual performance regressions for elite athletes’ personal best speeds (mph) in several events on the inverse hyperbolic cosecant (IHC) distance variables defined in column 1, row 4 of Table 1. The first term is *D* (distance) and the second is $log\left(1+\sqrt{1+D^{\text{2}}}\right)$. Greek letters refer to the formula in Table 1; “##” stands for “undefined.” The data come from Wikipedia [23].

The explanatory power of these regressions, as measured by the R-squared and associated F-test, is high for all five record holders. Moreover, the variables defined by Table 1 are statistically significant in most cases, despite the small sample sizes. The regression for Rudisha is an exception; the implicit value for the α parameter in the IHC formula is undefined. As shown in Fig 7, however, even this regression has a plausible predicted value. Overall, these results indicate that the IHC form closely approximates the relationship between speed and distance for world-record-setting runners, at least with sufficient personal-best observations.

**Fig 7:**
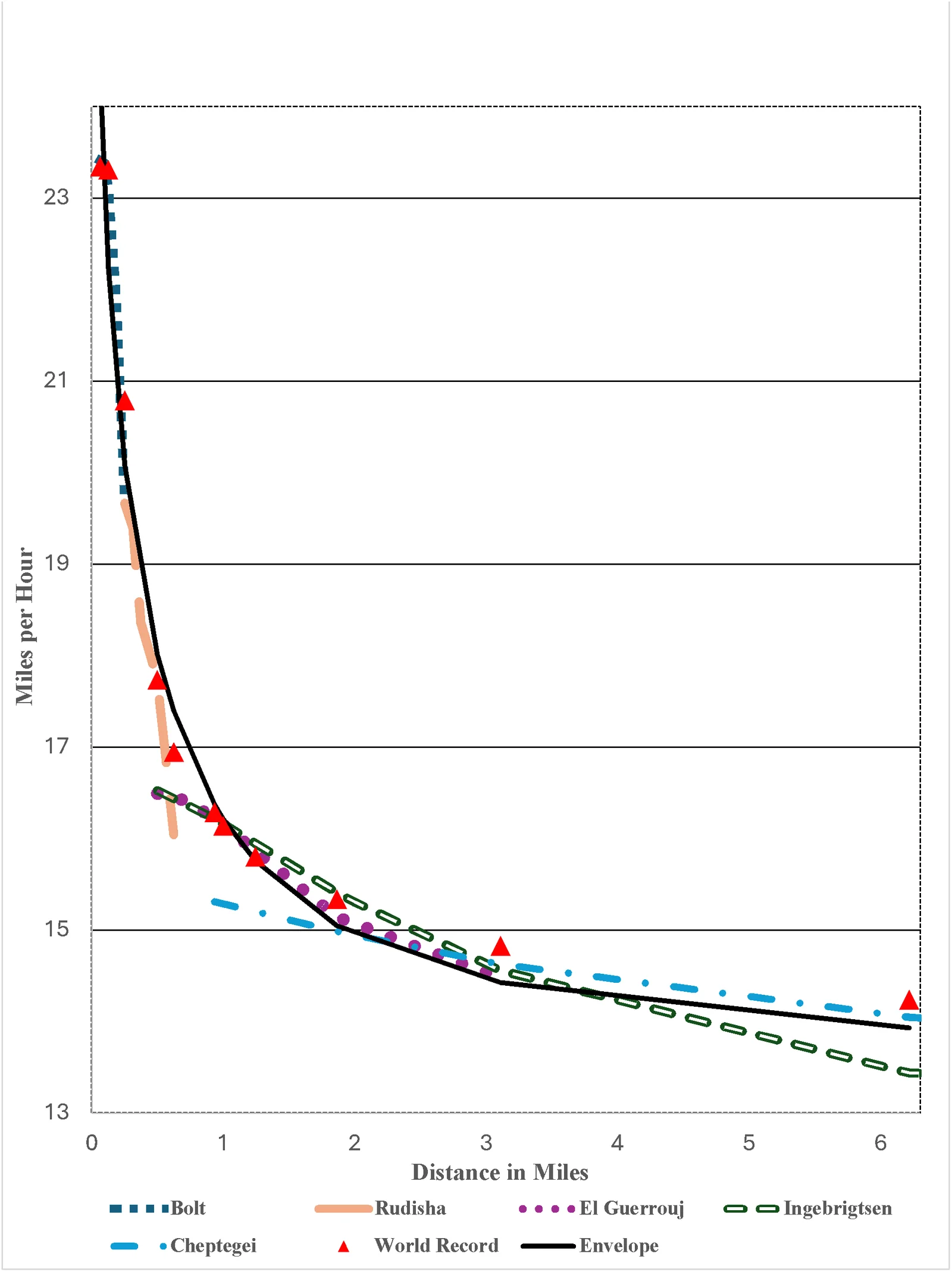
World records and individual performance curves, men’s runing, 2024.

Fig 7 demonstrates that the performance curves of elite runners tend to be close to linear, at least in the range of races in which they officially participate. As expected for these world-record holders, each of their individual performance curves is approximately tangent to the estimated world-record envelope. The performance curves of El Guerrouj and Ingebrigtsen are similar, but they differ enough so that El Guerrouj (curve with dots) holds the records for 1500 meters and the mile (1609.34 meters), whereas Ingebrigtsen (curve with ovals) holds the records for 2000 and 3000 meters. To keep Fig 7 legible, many worthy track stars are excluded, including Kelvin Kiptum and Michael Johnson, the 2024 record holders in the marathon and 400 meters, respectively.

These individual performance curves illustrate the speed-distance tradeoffs that face elite runners. In principle, individual performance curves could be extrapolated, as they are in the hypothetical individual performance curves of Fig 1, to races that are far from the distances in which a runner competes. However, this type of extrapolation suggests that elite runners who specialize in events shorter than 10,000 meters would exhibit running speeds below 12 mph (19.3 km/h) if they tried to run in longer events. This sounds implausible. A more reasonable interpretation is to say that the formula in Table 1 provides a close approximation for the racing speeds of elite athletes for the set of races in which they formally participate.

Fig 1 in Blythe and Király [12] appears to be the same as Fig 7. In fact, however, the figure in [12] plots individual performances and world records, whereas Fig 7 shows the estimated individual performance curves and associated world-record envelope. These two figures are obviously related but conceptually distinct. Another link between this paper and [12] is the recognition that the forms of the individual performance curves and the pattern of world records are linked. Although Blythe and Király [12] do not use the term “envelope,” they do argue [2, p. 12] that “both the power law and the broken power law on world record data can be understood as epiphenomena of the individual power law and its non-linear corrections.”

### 3.4. Testing hypotheses about human physiology

Many articles in the literature on world records interpret the shape of the world-record envelope as a reflection of human physiology. For example, Roy et al. [21, p. 357] write:

Running speed declines with increasing distance and age. Over long distances, where aerobic metabolism is dominant, speed declines in proportion to the logarithm of distance. Over short distances, anaerobic metabolism contributes significantly to performance, and speed is increased relative to the trend of the long-distance data.

This statement may apply to the world-record envelope, but it does not apply to the speed-distance tradeoff for individual runners. The results in Table 7, which are plotted in Fig 7, explain why this is true: the individual performance curves for all runners in this table and figure are close to linear. These results do not support the view that all runners shift from anaerobic metabolism to aerobic metabolism at approximately the same race distance. Instead, they suggest that each elite runner has a unique trade-off between anaerobic and aerobic metabolism that results in an approximately linear relationship between speed and distance for the races in which they participate.

In other words, the results in Fig 7 and Table 7 show that some elite runners attain incredibly high speeds in short races and other elite runners maintain lower speeds for incredibly long distances. These results do not indicate that elite runners shift from a relatively steep performance curve to a relatively flat one when they shift from shorter races to longer ones. They also do not deny that a shift from aerobic to anerobic metabolism might occur as race length declines. Instead, they simply show that the decline in speed as race distance increases is larger for runners who specialize in short races than for long-distance specialists.

## 4. Discussion

A world record in a racing event can be characterized as the highest average speed for an event over a given distance. In this paper I show that racing world records can be described as the mathematical envelope of the performance curves of elite racers. Moreover, this envelope can be closely approximated with a regression of winning speed as a particular function of event distance. This function is the inverse hyperbolic cosecant, which is described in Table 1.

These insights about racing world records facilitate an understanding of variation in the speed-distance tradeoff across events of different lengths and make it possible to compare this tradeoff across sports, over time, and between men and women. I find that the speed differences between short and long events are particularly large in running compared to swimming and speed skating. In addition, the shape of the world-record envelope is similar for men and women in all three of these sports and similar across time, at least for men’s running.

The analysis in this paper also shows that the form of the world-record envelope and the forms of the associated individual performance curves are mathematically linked. Any study that examines both this envelope and the associated individual performance curves needs to recognize this linkage. Studies that use the same form for both types of curves or that estimate curves using average values for a set of elite athletes are inconsistent with this lesson.

Regardless of its functional form, the world-record envelope, that is, the overall relationship between speed and distance, is steeper for short-distance races and flatter for long-distance races. The consensus in the literature is that this shape reveals a shift from anerobic physiology at short distances to aerobic physiology at long distances. However, the slope of the world-record envelope does not reveal the changing physiological demands on an individual racer as event distance increases. Instead, it reveals how much the winning speed declines as one moves from one world-record holder to another world-record holder in a longer event.

In the case of elite male runners, the individual performance curves, which are estimated with a runner’s personal best times, are close to linear, at least for the races a runner enters. Thus, these curves indicate that the high speeds of some elite runners in short races fall off rapidly as race length increases, whereas the more moderate speeds of some other elite runners show little drop-off as their race length goes up. These results are consistent with the consensus view that elite runners experience a gradual shift from anerobic to aerobic physiology as race length increases, but this shift is different for each runner.

Identifying the role of physiological factors requires a model that varies across a sample of athletes. One example is suggested by Blythe and Király [2, p. 13], who argue that their “results may have implications for existing hypotheses and findings in sports science on the differences in physiological determinants of long and short distance running respectively.”

The methods in this paper lead to an alternative approach. The first step is to pool the data for a set of elite athletes with multiple performances in a given sport, such as the data for world record holders in Table 7. Adding additional events for each athlete in the sample would increase the precision of the results. The next step is to add data for the relevant physiological traits of these athletes, such as their age, maximal oxygen intake, muscle fiber types, and training methods. Data could also be added for racing conditions, such as whether the race took place at the Olympics, the elevation gains or losses (for marathons and half marathons), and the quality of the ice (for skating).

These data would make it possible to run a baseline regression with the specification in Table 7 (or with some alternative individual performance curve) using these individual traits and racing conditions as additional explanatory variables. This type of regression would indicate whether individual performance curves shift up or down when these new variables are included. A possible result, for example, is the impact of maximal oxygen intake on racing speed. Moreover, interactions between these physiological and racing condition variables and the two variables defined by the regressions in Table 7 could indicate whether any of these new variables lead to changes in the shape of the individual performance curves. By interacting maximal oxygen intake with the two variables in Table 7, for example, one could determine whether higher maximal oxygen intake leads to a steeper individual performance curve.

The main contribution of this approach is that it leads to direct tests of hypotheses concerning the factors that influence racing performance. Although this approach may provide many insights in some circumstances, its applicability is limited to cases in which the physiological traits of individual racers and their speeds in many events can both be observed.

Overall, this paper builds on the concept of a mathematical envelope to shed light on the relationship between speed and distance in racing events. In addition, this paper draws on the concept of individual performance curves, which are related to envelopes, to provide a platform for future studies concerning the impact of elite racers’ physiological traits on their racing speeds.

## Supporting information

S1 Data WR data revised final. (XLSX)
